# It Is All in the Genes: A Story of Unexpected Survival in a 67-Year-Old Male with Metastatic Pancreatic Cancer

**DOI:** 10.1155/2023/8751205

**Published:** 2023-07-27

**Authors:** Patsy W. P. Lee, Scott W. Strum, Elena Tsvetkova

**Affiliations:** ^1^Department of Internal Medicine, Schulich School of Medicine and Dentistry, Western University, Canada; ^2^Department of Medical Oncology, London Regional Cancer Program, Western University, Canada

## Abstract

**Background:**

We describe a case report of a 67-year-old male with PDAC who experienced an exceptional survival outcome during systemic therapy and its implications in precision medicine. We hypothesize that his outcomes are attributable, in part, to a germline *BRCA2* deletion and somatic *GNAS* substitution.

**Methods:**

Retrospective single-patient chart review was performed at the London Regional Cancer Program, as well as a structured literature search spanning all years in PubMed of BRCA and GNAS mutations in pancreatic cancer.

**Results:**

The case described herein represents a 67-year-old male who survived over 27 months after third-line treatment with gemcitabine, docetaxel, and capecitabine (GTX) chemotherapy for metastatic PDAC after progression on gemcitabine and Abraxane and then on FOLFIRINOX. His survival far exceeded the median overall survival metrics. Genetic testing revealed a pathogenic heterozygous germline *BRCA2* 6643delT p.(Tyr2215Thrfs^∗^14) frameshift mutation and somatic GNAS 2531*G* > *A* p.(Arg844His) mutation.

**Conclusions:**

This case highlights the urgent need to expand our knowledge of cancer biology to advance personalized cancer treatment and therapy development.

## 1. Introduction

Pancreatic cancer is the fifth leading cause of cancer-related death in the United States [1]. Common mutations associated with it are *KRAS*, *TP53*, *CDKN2A*, and *SMAD4*. [2] Despite growing knowledge surrounding this disease, 5-year survival is approximately 11% [3, 4]. We describe a 67-year-old male who has strikingly lived 30 months since the start of third-line therapy for stage IV cancer despite rapid progression on first and second-line treatment. Initially, third-line treatment was paused due to toxicity, but his disease was stable for a year during this time. We hypothesize that his survival and lack of progression off-therapy are attributable, in part, to a germline *BRCA2* deletion and somatic *GNAS* substitution. Personalization of cancer management to the molecular level offers the precise understanding and prediction of disease biology and natural history.

## 2. Clinical Case

A 67-year-old male presented to medical attention in the summer of 2019 with unintentional weight loss of 40 pounds. His past medical history included type two diabetes, hypertension, hypogonadism, hepatic steatosis, and bilateral hearing loss. He had no family history of cancer. A contrast-enhanced CT scan demonstrated dilatation of the main pancreatic and common bile duct and mildly enlarged peripancreatic lymph nodes. An MRI identified a poorly circumscribed lesion in the pancreatic duct (1.5 × 2.0 × 2.9 cm). Fine needle core biopsy revealed neoplastic cells, concerning for adenocarcinoma. The patient underwent a Whipple procedure in October 2019, with pathology revealing a grade 1, 5.1 cm adenocarcinoma of the pancreatic head, with 0/15 lymph nodes involved, and negative margins, consistent with pT3N0 disease. Preoperative CA19-9 was 37 U/mL (ULN 34 U/mL).

**Figure 1:**
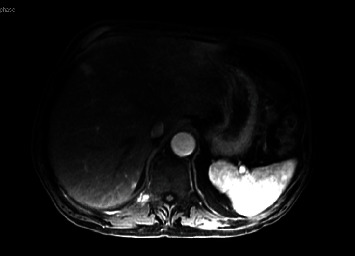
MR image of the liver lesion prior to third-line GTX therapy.

In December 2019, prior to starting adjuvant chemotherapy, MRI confirmed the presence of two liver masses (14 and 4 mm) alongside positive portocaval lymph nodes. Serum CA19-9 was 54 U/ml (Table 1). The patient was started on palliative gemcitabine and Abraxane (GA) in January 2020. After three cycles, a CT scan demonstrated disease progression. He was then treated with five cycles of FOLFIRINOX (5-fluorouracil, leucovorin, irinotecan, and oxaliplatin) in the second line starting in April 2020. Five months later, imaging demonstrated progression (Figure 1). Third-line GTX (gemcitabine, docetaxel, and capecitabine) was initiated. Due to clinical decline, chemotherapy was suspended after the second cycle in August 2020. Referral was made to palliative care in September 2020.

From September 2020 to August 2021, 11 months of stable disease were captured on surveillance imaging. During this time, he had a significant improvement in his performance status, alongside a decrease in his CA19-9 (Table 1). CT scan identified progression in August 2021, but GTX was restarted as per protocol when the patient became symptomatic in October 2021.

Somatic tumor genetic testing in December 2021 from his Whipple's resection revealed a Tier 1 *BRCA2* mutation and Tier 2 *GNAS* mutation. Germline testing in February 2022 identified the presence a *BRCA2* mutation that matched somatic testing. Unfortunately, due to progression on platinum-containing therapy, he was ineligible for PARP inhibitor treatment. Thus, he was continued on GTX with brief treatment suspensions for intercurrent illness until disease progression in November 2022. He was started on fourth-line gemcitabine and cisplatin in December 2022 and remains on this up to and including January 3, 2023. The patient has since retained a performance status of 0-1 and good symptom control up to this date.

## 3. Discussion

Herein, we present a patient with metastatic pancreatic adenocarcinoma (PDAC) who demonstrated disease progression on GA and FOLFIRINOX but experienced a sustained response on GTX. He has lived for 30 months after initiation of this third-line treatment, with 11 of those months off-chemotherapy with disease stability. The 18-month survival for patients with stage IV PDAC is approximately 18.6% [5]. Patients with metastatic PDAC who were treated with GTX had an overall median progression-free survival (mPFS) and median overall survival (mOS) of 6.3 and 11.2 months for responders, respectively [6]. Treatment of metastatic pancreatic cancer with FOLFIRINOX in the first-line setting had a mOS of 11.1 months [5]. With GA, patients had a mOS of 8.5 months and mPFS of 5.5 months in the first-line setting [7]. Although this patient did not tolerate FOLFIRINOX, his OS and PFS have surpassed that of patients on GA and GTX combined.

A possible explanation for his excellent survival outcome may lie in his underlying biology. Somatic tumor genetic testing revealed a heterozygous Tier 1 [8] *BRCA2* 6643delT p.(Tyr2215Thrfs^∗^14) pathogenic frameshift mutation resulting in premature termination of the BRCA2 protein, alongside a Tier 2 GNAS 2531*G* > *A* (Arg844His) mutation. Germline testing confirmed the presence of the same *BRCA2* mutation. *BRCA1/2* are tumor suppressor genes that repair double-strand breaks in DNA to prevent tumor development. Germline *BRCA1/2* variants have clinical implications. Thirty cancer types have been identified in people with *BRCA1/2* variants [9]. In a Japanese cohort of 63,000 patients, its presence was associated with an increased risk of female and male breast, gastric, ovarian, pancreatic, prostate, and esophageal cancers [10]. Furthermore, patients with tier I mutations in the *BRCA2* gene have an odds ratio (OR) of 6.20 of developing PDAC. PDAC patients with tier I mutations in *BRCA2* alone were significantly associated with an earlier age of PDAC diagnosis (60.5 years vs. 63.3 years, *p* = 0.01) and family history of breast cancer (OR 2.07, adjusted *p* = 0.04) [11].

From a prognostic perspective, no differences for surgically resectable *BRCA*-associated PDAC were seen in a retrospective case-control study compared to sporadic PDAC [12]. Treatment efficacy, however, can be impacted. The phase III POLO study demonstrated a significant PFS benefit for active olaparib maintenance therapy versus placebo for patients with a germline *BRCA1/2* mutation whose disease had not progressed after more than 16 weeks of first-line platinum-based chemotherapy (mPFS 6.7 months and 3.7 months, respectively (HR, 0.49; 95% CI, 0.33 to 0.73; *p* = 0.0004)) [13]. Furthermore, *BRCA1/2*-mutated cancers tend to exhibit sensitivity to platinum-based chemotherapy, demonstrating a higher mOS in patients treated with platinum agents compared to nonplatinum chemotherapies (22 vs. 9 months, *p* = 0.039) [14].

This patient also had a somatic *GNAS* mutation identified from tumor tissue. These mutations have been reported in up to 20% of human solid tumors [15]. However, its clinical significance is unclear. It is postulated that this mutation influenced the patient's clinical course through dysregulation of GNAS, its interaction with the *BRCA2* mutation, or a different mechanism. Given his exceptional survival outcome, more data is urgently needed to enhance patient outcomes through the use of personalized medical technologies.

Limitations to the report include a small body of knowledge to compare this outcome against, regarding the mutations found. In addition, his prolonged survival may have been accounted for, in part, through low disease burden initially. This may have been a confounder for his excellent survival. However, this does not necessarily account for his disease stability post-GTX. Finally, somatic and germline whole genome sequencing was not completed and may reveal information about genomic changes that may explain his natural history on therapy beyond the known mutations.

Ultimately, personalization of oncological treatment (known as precision cancer medicine (PCM)) has led to improvements in prognostication and therapeutic access. Pembrolizumab has been approved for use in patients with solid malignancies that demonstrate high microsatellite instability, with an ORR rate of 18.2% among PDAC patients in a pivotal basket trial [16]. For patients with *NTRK* fusion-positive tumors, entrectinib achieved objective responses in two of three PDAC patients in an integrated analysis of three phase I/II trials [17]. As previously mentioned, maintenance olaparib after platinum-based induction chemotherapy in germline *BRCA* mutant advanced PDAC showed superior mPFS compared to placebo [13].

These benefits have been explored outside of pancreatic cancer as well. Providing breast cancer patients with targeted therapies compared to maintenance treatment improved PFS when genomic alterations were classified as level I/II according to the ESMO scale for clinical actionability of molecular targets (ESCAT) (HR of 0.41, *p* < 0.001) [18]. In a 2017 retrospective analysis, PCM was found to almost double the average PFS in metastatic patients matched to various demographics (22.9 vs. 12.0 weeks, *p* = 0.002), without significant change in cost [19]. In advanced endometrial cancer patients, those who received matched targeted therapy based on comprehensive genomic profiling led to an objective response or stable disease with a median treatment duration of 14.6 months in 62.5% of patients [20]. Lastly, in Canada, the Ontario-wide Cancer Targeted Nucleic Acid Evaluation (OCTANE) trial aims to develop a province-wide registry of NGS testing to evaluate its outcomes and cost to inform broader indications for NGS in advanced cancer due to a low enrolment rate for targeted therapies (4-32%) [21]. A limitation to PCM is lowered efficacy in heavily pretreated patients, due to the development of molecular resistance [22].

Overall, NGS offers a personalized approach to cancer management. Alterations in the genome can modify the clinical course of localized and metastatic disease [23]. We hypothesize that this patient's germline *BRCA2* mutation and somatic *GNAS* mutation constituted important components of his biological landscape that led to his remarkable 30-month sustained response to third-line GTX (including 11 months off-therapy). It is hoped that with the expansion of knowledge in cancer genetics, we may better understand how genetic variants affect the natural history of the disease, allowing for improved discovery and use of targeted treatments.

## Figures and Tables

**(a):** 

Year	2019				
Month	Aug	Sept	Oct	Nov	Dec
CA19-9	37	—	—	—	54
Treatment	NT	NT	NT	NT	NT

**(b):** 

Year	2020											
Month	Jan	Feb	Mar	Apr	May	June	July	Aug	Sept	Oct	Nov	Dec
CA19-9	44	—	52, 56	—	61	59	62	—	118	126	—	—
Treatment	GA	GA	GA	FFX	FFX	FFX	GTX	GTX	NT	NT	NT	NT

**(c):** 

Year	2021											
Month	Jan	Feb	Mar	Apr	May	June	July	Aug	Sept	Oct	Nov	Dec
CA19-9	—	—	—	—	59	—	—	99	—	81	—	131, 146
Treatment	NT	NT	NT	NT	NT	NT	NT	NT	NT	GTX	GTX	GTX

**(d):** 

Year	2022											
Month	Jan	Feb	Mar	Apr	May	June	July	Aug	Sept	Oct	Nov	Dec
CA19-9	84	117	87, 135	86	54	192	282	64	78	—	—	—
Treatment	GTX	GTX	GTX	GTX	GTX	NT	NT	NT	NT	GTX	GTX	GC

NT: no treatment; GA: gemcitabine, abraxane; FFX: FOLFIRINOX: 5-fluorouracil, leucovorin, irinotecan, oxaliplatin; GTX: gemcitabine, docetaxel, capecitabine; GC: gemcitabine, cisplatin.

## Data Availability

The data used to support the findings of this study are included within the article.
